# Characteristic of new *Phaffia rhodozyma* yeast strains isolated from birch slime fluxes in Poland

**DOI:** 10.1007/s00203-024-04161-7

**Published:** 2024-10-16

**Authors:** Anna M. Kot, Katarzyna Pobiega, Marek Kieliszek, Katarzyna Michalak, Stanisław Błażejak

**Affiliations:** 1 Department of Food Biotechnology and Microbiology, Institute of Food Sciences, Warsaw University of Life Sciences, Nowoursynowska 159C, Warsaw, 02-776 Poland; 2 Department of Epizootiology and Clinic of Infectious Diseases, Faculty of Veterinary Medicine, University of Life Sciences in Lublin, Głęboka 30, Lublin, 20-612 Poland

**Keywords:** Strain characterization, *Phaffia rhodozyma*, Carotenoids, MALDI-TOF MS

## Abstract

Three new strains of *Phaffia rhodozyma* yeast have recently been isolated in Poland. The aim of this study was to phenotypically characterize these strains and to compare them with the properties of the reference strain. The potential for carotenoid biosynthesis in these strains was also determined, depending on temperature, carbon, and nitrogen sources in the medium. *Phaffia rhodozyma* yeasts were also identified by MALDI-TOF MS. There were minor differences in cell morphology among the strains. All strains reproduced asexually by budding and formed spherical chlamydospores. No ability for sexual reproduction was observed. Physiological tests showed minor variations between the reference strain and the isolates, likely due to the geographical specificity of the habitat from which they were originally isolated. Analysis of protein spectra showed that the tested yeast isolates had seven common peaks of different intensities, with masses at 2200, 2369, 3213, 3628, 3776, 3921, and 4710 m/z. Moreover, additional strain-dependent spectra were found. The amount of synthesized carotenoids varied with the carbon and nitrogen sources used, as well as the temperature. The best producer of carotenoids was the *P. rhodozyma* CMIFS 102 isolate.

## Introduction

In spring, many species of deciduous trees secrete juices from injured parts. This fluid is colonized with microorganisms, including yeast. It has a viscous, thick consistency and various colors. At the end of the 19th century, Ludwig first described this exudate. According to his description, its color resulted from the intensive growth of an organism with a yeast-like structure, which he named *Rhodomyces dendrorhous* (Wettstein 1985; Weber 2006).

In 1967–1968, Phaff’s team isolated ten new strains of yeast with red colonies from the sap of deciduous trees (mainly *Betula*, *Alnus*, and *Fagus* species) growing in mountainous regions of Japan, on the islands of Hokkaido and Honshu, and in Alaska (Kucsera et al. 2000). Due to its origin and orange-red color, this yeast was initially called *Rhodozyma montanae* (Johnson 2003). However, its unique features, along with the lack of a Latin description, led to a change in the genus name to *Phaffia*, also to commemorate its discoverer, the Dutch scientist Herman Jan Phaff (Miller et al. 1976). Golubev concluded that *Phaffia rhodozyma* is the anamorphous state of the teleomorphic yeast *Xanthophylomyces* and a synonym for *R. dendrorhous*, as originally described by Ludwig (Golubev 1995).

Since the first isolation of *P. rhodozyma*, several other isolates have also been obtained from the sap of spring deciduous trees of the genera *Betula*,* Alnus*,* Fagus*, and *Cornus*. These trees were located in the Northern Hemisphere, mainly in Germany, Finland, and Russia (Weber 2006). Given the geographical distribution of trees from which *Phaffia* yeast was isolated, it was initially assumed that this species occurs only in the Northern Hemisphere (Golubev 1995). However, at the beginning of the 21st century, it was also isolated and identified in Italy (Weber and Davoli 2005), and in subsequent years in Argentina (Libkind et al. 2007; Contreras et al. 2015) and Chile (Weber et al. 2008). Most isolates obtained so far have come from the sap of deciduous trees, with the exception of yeast isolated in Argentina from the mycelium of *Cyttaria hariotti* growing on *Nothofagus dombeyi* trees (Libkind et al. 2007) and a strain isolated in Chile from the surface of *Eucalyptus globulus* leaves (Weber et al. 2008). Recently, three new strains of *P. rhodozyma* yeast have been isolated from spring slime fluxes of birch (*Betula pendula*) in Poland (Kot et al. 2024). According to (Bellora et al. 2016), the unique biotechnological properties of *P. rhodozyma* result from its adaptation to life in association with plant matrices, especially tree exudates. These properties also depend on the geographic location and the isolation environment, including tree hosts (David-Palma et al. 2014). This yeast is considered an excellent producer of astaxanthin (Pan et al. 2017).

Astaxanthin is a 3,3′-dihydroxylated and 4,4′-diketolated derivative of β-carotene with strong antioxidant properties. The second important pigment produced by the yeast *P. rhodozyma* is β-carotene (Schmidt et al. 2010). Animals lack the ability to produce carotenoids de novo and can obtain them through their diet. Moreover, organisms bred in aquaculture, such as salmon, trout, and shrimp, are deprived of their natural food sources, such as algae or plankton, which provide these pigments. Therefore, their feed must be supplemented with carotenoids to get benefit from the healthy effects of carotenoids. The addition of *P. rhodozyma* yeast to the feed also ensures proper coloration in cultured fish and marine crustaceans (Kucsera et al. 2000; Johnson 2003). Additionally, it positively impacts animal health (Elwan et al. 2019) and enhances the color of egg yolks in poultry feed, making them more attractive to consumers (Zhu et al. 2021). The use of these microorganisms in the pharmaceutical industry is related to the properties of the carotenoids they produce, which can counteract cell aging and potentially delay or prevent degenerative changes (Schmidt et al. 2010). Another application is the production of enzymes, such as β-amylase (EC 3.2.1.2), which breaks down starch and other polysaccharides (Díaz et al. 2003) and is used, for example, as a digestive aid (Ray and Nanda 1996). Additionally, β-fructofuranosidase (EC 3.2.1.26) is responsible for producing neo-fructo-oligosaccharides (Gimeno-Pérez et al. 2015).

The aim of this study was to examine the morphological and physiological properties of three *P. rhodozyma* yeast strains isolated from spring slime fluxes of birch (*B. pendula*) in Poland and to compare them with the properties of the reference strain. Additionally, the study assessed the ability of these yeasts to biosynthesize carotenoids in various media and at different culture temperatures. Yeasts were also identified by MALDI-TOF MS.

## Materials and methods

### Biological material

Three new *P. rhodozyma* yeast strains were isolated from spring slime fluxes of birch (*B. pendula*) in our previous work (Kot et al. 2024). The strains were stored in the Collection of Microorganisms of the Institute of Food Science (CMIFS) at the Warsaw University of Life Sciences. The yeast *P. rhodozyma* DSM 5626 (CBS 5905) obtained from the Leibniz Institute DSMZ was used as a reference strain.

### Identification based on ITS regions

DNA was isolated using the chloroform-phenol method as described in the previous work (Kot et al. 2024). The PCR reaction mixture contained primers (20 pmol each), dNTPs (0.2 mM), MgCl2 (1.5 mM), Taq polymerase (1 U). 1 µl of DNA solution at a concentration of 250–300 ng/µL was added to the mixture. Primers ITS1 (5′-TCCGTAGGTGAACCTGCGG-3’) and IST4 (5′-TCCTCCGCTTATTGATATGC-3’) were used. The PCR process was performed using the following parameters: initial denaturation at 94 °C (4 min), 30 cycles: denaturation at 94 °C (30 s), annealing at 55 °C (30 s), extension at 72 °C (1 min), and at the end of the process, a single final extension at 72 °C for 4 min was applied (Fujita et al. 2001). PCR samples were sent to Genomed (Warsaw, Poland) for Sanger sequencing. Sequences were analyzed using Chromas Lite (version 2.6.6.) and then compared to sequences deposited at the National Center for Biotechnology Information. Based on these results, ITS sequences were deposited in GenBank under the following accession numbers: *P. rhodozyma* CMIFS 102 (accession number OR616623), *P. rhodozyma* CMIFS 110 (accession number OR616628) and *P. rhodozyma* CMIFS 114 (accession number OR616635).

### MALDI-TOF identification of yeast

Yeast strains were characterized using an UltrafleXtreme MALDI-TOF mass spectrometer (Bruker Daltonics, Germany). A standard ethanol/formic acid extraction procedure, following the manufacturer’s instructions, was used. First, several yeast colonies from a pure culture were transferred to an Eppendorf tube, suspended in 150 µL of MQ water, vortexed for 1 min, and centrifuged for 2.5 min at 13,000×g. This step was repeated five times. The yeast pellet was then covered with 450 µL of pure ethanol (≥ 99.8%) and mixed thoroughly by vortexing for 1 min. After centrifugation for 2.5 min at 13,000×g, the supernatant was discarded, and the yeast pellet was subjected to protein extraction with 40 µL of 70% formic acid. After 1 min of vortexing, 40 µL of ≥ 99.9% acetonitrile was added, and the sample was mixed again by vortexing. Finally, the sample was centrifuged for 2.5 min at 13,000×g, and 1 µL of the obtained supernatant was spotted in triplicate on the MTP AnchorChip MALDI metal plate (Bruker Daltonics, Germany). The dried sample was covered with 1 µL of HCCA matrix solution (10 mg of α-Cyano-4-hydroxycinnamic acid dissolved in 50% acetonitrile, 47.5% ultrapure water, and 2.5% trifluoroacetic acid) and left to dry at room temperature. The MALDI plate was then transferred to the UltrafleXtreme MALDI-TOF mass spectrometer, and FlexControl 3.4 software (Bruker Daltonics, Germany) was used for automatic collection of spectra in the range of 2000–20,000 Da. The obtained spectra were compared to the reference strain (*P. rhodozyma* DSM 5626) using MALDI Biotyper 3.1 software (Bruker Daltonics, Germany). The results were reported for each sample, along with confidence scores ranging from 0.00 to 3.00. The following score values, as proposed by the manufacturer, were used for interpreting results: a log (score) < 1.70 indicates no reliable identification (−); a log (score) of 1.70–1.99 allows identification at the genus level (+); a log (score) of 2.00–2.29 indicates highly probable identification at the genus level and probable at the species level (++); and a log (score) ≥ 2.30 indicates highly probable identification at the species level (+++).

### Yeast morphology

The yeast was inoculated onto the following media: (a) YPD agar medium (glucose 2%, peptone 2%, yeast extract 1%, agar 2%; pH 5.6); (b) xylitol agar medium (xylitol 0.5%, agar 2%; pH 5.6); (c) agar medium with ribitol (ribitol 0.5%, agar 2%; pH 5.6); (d) YM agar (yeast extract 0.3%, malt extract 0.3%, peptone 0.3%, glucose 1%, agar 2%; pH 5.6); and (e) CornMeal Agar (DIFCO). The yeast was grown at 20 °C for 14 days. Additionally, the ability to produce spores was assessed on Remel Ascospore Agar (potassium acetate 1%, yeast extract 0.25%, glucose 0.1%, agar 2%; pH 5.6) and incubated at 20 °C for 30 days.

### Yeast physiology

The ability to ferment D-glucose, D-fructose, D-galactose, D-sucrose, D-lactose, D-maltose, D-trehalose, D-raffinose, and inulin was determined. The medium contained 2% of the tested compound, 1% peptone and 0.5% yeast extract. Incubation lasted 5 days at 20 °C. Fermentation tests was carried out under static conditions. The ability to assimilate the following carbon sources was tested: D-glucose, glycerol, calcium 2-keto-gluconate, L-arabinose, D-xylose, adonitol, xylitol, D-galactose, inositol, D-sorbitol, methyl-α-D-glucopyranoside, N-acetylglucosamine, D-cellobiose, D-lactose, D-maltose, D-sucrose, D-trehalose, D-melezitose, D-raffinose, D-fructose, DL-lactic acid, citric acid, D-mannitol, erythritol, and inulin. For this purpose, Yeast Nitrogen Base (Merck Millipore) medium and a 2% addition of the tested carbon source were used. Incubation lasted 5 days at 20 °C. The ability to assimilate nitrogen compounds such as urea, potassium nitrate, and ammonium sulfate was also tested. Yeast Carbon Base (Merck Millipore) was used. This medium was supplemented with 0.5% of the tested nitrogen source. The yeast was incubated at 20 C for 5 days. Assimilation tests was carried out under static conditions. The yeast growth range was examined using a liquid YPD medium (2% glucose, 2% peptone, 1% yeast extract) with initial pH values of 1.0, 2.0, 3.0, 4.0, 5.0, 6.0, 7.0, 8.0, 9.0, 10.0, 11.0, and 12.0. Additionally, the yeast’s growth at high concentrations of glucose (5%, 10%, 20%, 30%, 40%, 50%) and sodium chloride (0.5%, 1%, 2%, 5%, 7.5%, 10%, 15%) was determined. For all these tests, incubation was performed at 20 °C for 5 days in shaken conditions. The growth temperature range of the tested strains was also determined (Pincus et al. 2007; Devadas et al. 2017).

### Enzymatic properties

The ability to produce catalase (tested with H₂O₂), urease (tested with urea), and hydrolyze gelatin, as well as to produce lipolytic enzymes (tested with tributyrin and Tween 20), and synthesize amylolytic (tested with starch) and cellulolytic enzymes (tested with carboxymethylcellulose) was determined. In order to test the ability to produce catalase, hydrogen peroxide was dripped onto the yeast colonies. The appearance of gas bubbles indicated a positive result. A test for urease production was performed similarly, using a 2% urea solution. For the gelatin liquefaction test, a medium with the following composition was used: NaCl 0.5%, tryptone 1%, beef extract 0.3% and gelatin 12% (Sun et al. 2024). Two media were used to study lipolytic activity. The first one contained peptone 0.5%, yeast extract 0.3%, agar 2%, and tributyrin 0.5% (Lima et al. 1991). The composition of the second medium was as follows: peptone 1%, yeast extract 0.3%, NaCl 0.5%, CaCl_2_ 0.01%, agar 2%, Tween 80 0.5% (Aktaz et al. 2002). After inoculation, the plates were incubated for 5 days at 20 °C. A positive result on the Tween 80 medium was the appearance of a precipitate around the colony. This was the result of the precipitation of calcium salts, which were formed as a result of the reaction of fatty acids released by extracellular lipases. In the case of the tributyrin medium, a positive result was indicated by the appearance of clear zones around the colony. To determine the ability to degrade starch, a medium with the following composition was used: peptone 1%, starch 0.2%, agar 2%. After 5 days of incubation at 20 °C, Lugol’s solution was dropped onto the surface of the plate (Kwon et al. 2020). To determine cellulolytic activity, the following medium was used: carboxymethylcellulose 1%, Na_2_CO_3_ 1%, peptone 0.5%, yeast extract 0.5%, NaCl 0.5%, KH_2_PO_4_ 0.1%, MgSO_4_∙7H_2_O 0.2%, agar 2%. Cellulolytic activity was indicated by the clear zones around the colony after the Congo red solution had been added (Fu et al. 2022).

### Yeast cultures for carotenoid biosynthesis

The medium used to select the optimal temperature for carotenoid biosynthesis contained 30.0 g/L glucose, 5 g/L (NH_4_)_2_SO_4_, 5.0 g/L yeast extract, 1.5 g/L KH_2_PO_4_, 1.0 g/L MgSO_4_ × 7 H_2_O, 0.15 g/L CaCl_2_, 0.1 g/L NaCl, 0.1 g/L MnSO_4_ × H_2_O, 0.1 g/L ZnSO_4_ × 7 H_2_O, and 0.1 g/L CuSO_4_ × 5 H_2_O. The pH value of the medium was 5.6.

The media used to select the carbon source for biosynthesis contained 30.0 g/L of the carbon source (glucose, maltose, xylose, sucrose, glycerol, mannitol, molasses, or fructose), 5.0 g/L (NH_4_)_2_SO_4_, 5.0 g/L yeast extract, 1.5 g/L KH_2_PO_4_, 1.0 g/L MgSO_4_ × 7 H_2_O, 0.15 g/L CaCl_2_, 0.1 g/L NaCl, 0.1 g/L MnSO_4_ × H_2_O, 0.1 g/L ZnSO_4_ × 7 H_2_O, and 0.1 g/L CuSO_4_ × 5 H_2_O.

The media used to select the nitrogen source for biosynthesis contained 30.0 g/L glucose, 5.0 g/L of a nitrogen source (ammonium sulfate, peptone, diammonium hydrogen phosphate, ammonium chloride, urea, or casein hydrolyzate), 5.0 g/L yeast extract, 1.5 g/L KH_2_PO_4_, 1 g/L MgSO_4_ × 7 H_2_O, 0.15 g/L CaCl_2_, 0.1 g/L NaCl, 0.1 g/L MnSO_4_ × H_2_O, 0.1 g/L ZnSO_4_ × 7 H_2_O and 0.1 g/L CuSO_4_ × 5 H_2_O.

Cultures were performed in Erlenmeyer flasks (500 mL) containing 100 mL of the appropriate liquid medium. To determine the optimal temperature for carotenoid biosynthesis, cultures were incubated at 8, 12, 16, 18, 20, 22, and 24 °C at 160 rpm for 120 h. Flasks containing media with various carbon and nitrogen sources were shaken at 140 rpm at 20 °C for 120 h as well (Eppendorf Innova 44, Germany).

### Biomass yield

Ten milliliters of culture were taken and centrifuged at 5000×g for 10 min (Centrifuge 5804R, Eppendorf, Germany). The supernatant was removed, and 10 mL of sterile distilled water was added to the wet biomass. The mixture was then centrifuged again using the same parameters. The sediment was dried at 85 °C, and the biomass yield was recorded in grams of dry matter (g_d.m._) per liter of the medium.

### Carotenoid content in yeast biomass

To determine the carotenoid content in yeast biomass, 1.5 mL of liquid cultures were centrifuged at 3000×g for 10 min (Centrifuge 5804R, Eppendorf, Germany), and the supernatant was removed. The wet biomass was washed with distilled water and centrifuged again. Next, 0.5 g of 500 μm glass beads and 2 mL of dimethyl sulfoxide were added to the wet biomass, mixed, and shaken on a rotator at 70 rpm for an hour (Multi BioRS-24, Biosan, USA) to break down the cell walls of the yeast cells. After this, 2 mL of acetone, petroleum ether (with 0.25% BHT), and 20% sodium chloride were added to the samples and shaken for an additional hour. To separate the phases of the mixture, the samples were centrifuged at 3000×g for 10 min. The absorbance of the ether phase was measured at 474 nm. The total carotenoid content, expressed as astaxanthin, was calculated using the formula: (µg/g_d.m._) = (*A* × *B* × 10000)/(*C* × *D*), where *A* is the volume of petroleum ether used to extract carotenoids, *B* is the absorbance at wavelength 474 nm, *C* is the 1% astaxanthin extinction coefficient (2100), and *D* is the dry biomass of extracted yeast cells (Choi et al. 2007).

### Statistical analysis

Statistical analysis was performed using R Project for Statistical Computing (version 8.9). One-way analysis of variance (ANOVA) and Tukey’s test were performed at a significance level of 0.05.

## Results and discussion

### MALDI-TOF mass spectra

Due to the lack of a spectrum standard in the MALDI-TOF MS system database, the protein profiles of *P. rhodozyma* birch isolates were compared with the DSM 5626 collection strain. The degree of similarity to the reference spectrum is represented by the score value (Table 1). The *P. rhodozyma* CMIFS 110 strain exhibited high similarity to the reference strain, with score values ranging from 2.025 to 2.099. For the CMIFS 114 isolate, the score values were slightly lower, ranging from 1.901 to 1.917. The lowest similarity index to the *P. rhodozyma* DSM 5626 strain was recorded for the CMIFS 102 strain, with values ranging from 1.708 to 1.794. Score values above 2.0 are considered indicative of correct identification at the species level. According to (Wieser et al. 2012) values between 2.0 and 1.7 represent reliable identification at the genus level. Chao et al. (2014) suggest that the MALDI-TOF MS system can be effectively used to identify various yeast species. Zhang et al. (2020) argue that MALDI-TOF MS is a suitable method for detecting small changes in peak spectra of microorganisms, such as *Candida californica*,* Metschnikowia pulcherrima*, and *Pichia kluyveri*, while accurately clustering strains at the species level.

**Table 1:** Results of yeast identification after automatic comparison of the spectra with the MALDI-TOF database of the reference strain

Analyte name	Rank (Quality)	Reference organism	Score value
*Phaffia rhodozyma* CMIFS 102	1 (+) 2 (+) 3 (+)	*Phaffia rhodozyma* DSM 5626	1.794 1.708 1.743
*Phaffia rhodozyma* CMIFS 110	1 (++) 2 (++) 3 (++)	*Phaffia rhodozyma* DSM 5626	2.063 2.025 2.099
*Phaffia rhodozyma* CMIFS 114	1 (+) 2 (+) 3 (+)	*Phaffia rhodozyma* DSM 5626	1.901 1.904 1.917

(+) – high similarity to the reference strain; (++) – average similarity to the reference strain

**Fig. 1:**
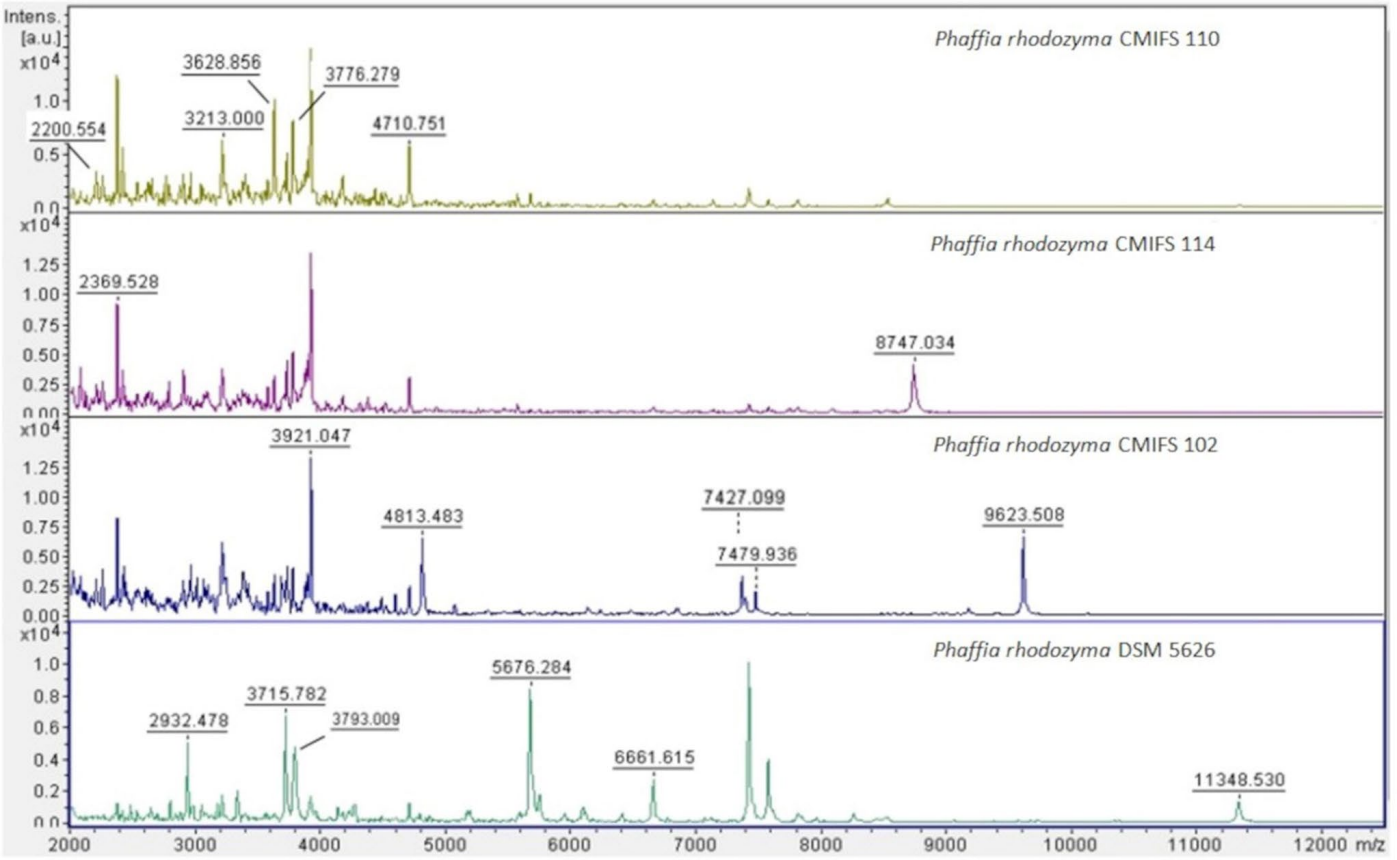
Spectra of yeast isolates and the reference strain produced by MALDI-TOF MS. The ion intensities and masses are shown on the Y and X axes, respectively. The m/z value is the mass-to-charge ratio

Individual protein bands differed in intensity between yeast strains (Fig. 1). All mass spectra of the tested yeast isolates showed good resolution of the obtained peaks, resulting in distinct protein profiles.

The tested yeast isolates exhibited seven common peaks of different intensities, with masses at 2200, 2369, 3213, 3628, 3776, 3921, and 4710 m/z. Additionally, the spectrum of the *P. rhodozyma* strain CMIFS 102 was characterized by three peaks at 7427, 7479, and 9623 m/z, which were not found in the other yeast strains (CMIFS 110, CMIFS 114). The masses 7427 and 7479 m/z were also observed in the strain *P. rhodozyma* DSM 5626. The spectrum of the *P. rhodozyma* CMIFS 114 strain displayed a unique peak at 8747 m/z, absent in the other strains. Bellora et al. (2016) indicate that genetic heterogeneity within *P. rhodozyma* may result in the presence of different peaks in protein spectra. This heterogeneity could be due to the yeast’s adaptation strategies for surviving under stressful conditions, such as UV light and oxidative damage. Using the MALDI-TOF MS technique for the identification of various microorganisms allows for the acquisition of mass spectra that reflect the protein profiles of the analyzed yeast. However, accurate identification is only possible for protein profiles with sequences available in the database. Given that the analyzed isolates were also identified as *P. rhodozyma* based on LSU (Kot et al. 2024) and ITS regions (this work), it is recommended that the MALDI-TOF MS system database be expanded to include spectra of new strains to increase the accuracy of identification.

### Morphology

**Fig. 2:**
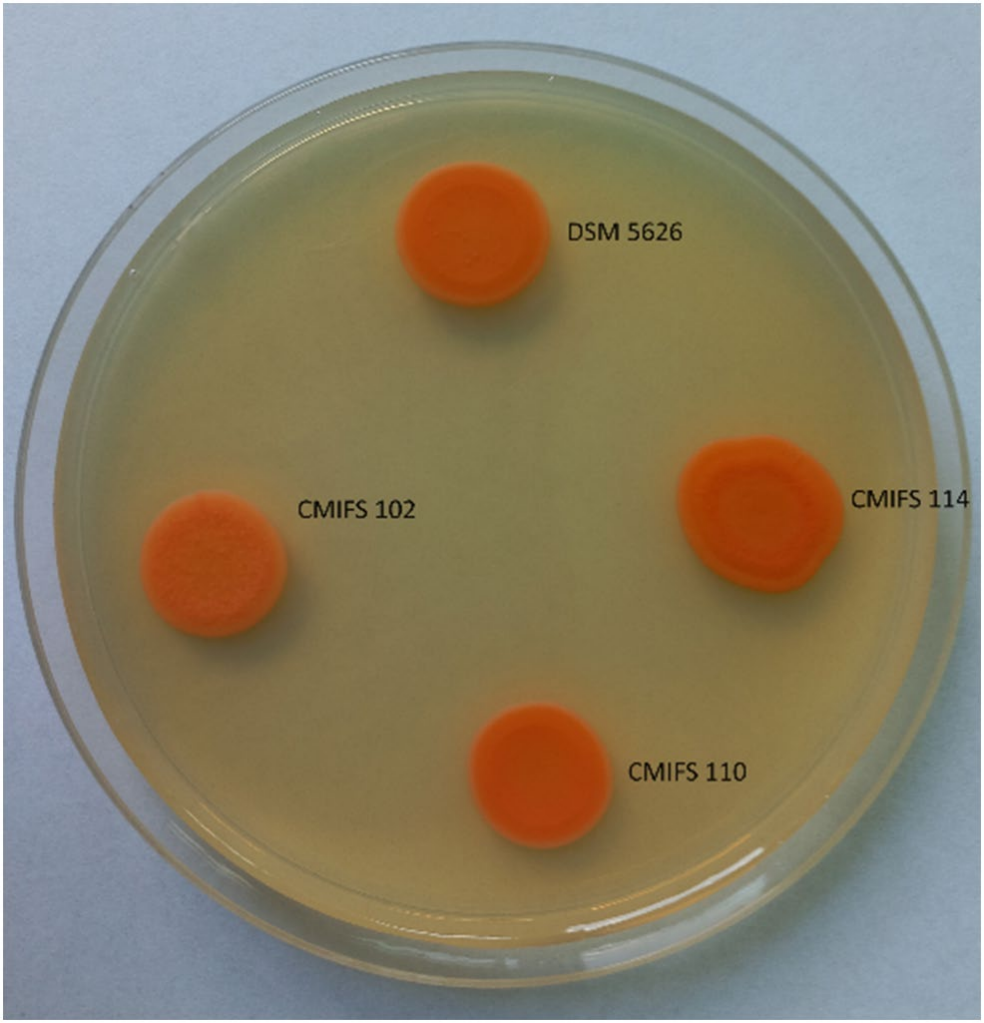
Morphology of *Phaffia rhodozyma* strains after 14 days of incubation at 20 °C on YM agar medium

The morphological characteristics of strains CMIFS 102, 110, and 114 were compared with the reference strain (*P. rhodozyma* DSM 5626) and showed no significant differentiation. After cultivation on YM agar, the colonies of all strains appeared orange (Fig. 2). The differences were limited to color intensity, with the CMIFS 114 strain exhibiting the most intense orange color. In contrast, the CMIFS 102 strain’s colonies were noticeably less intense. All colonies had a smooth surface and regular edges, except for the CMIFS 114 strain, which had wavy colony edges.

**Fig. 3:**
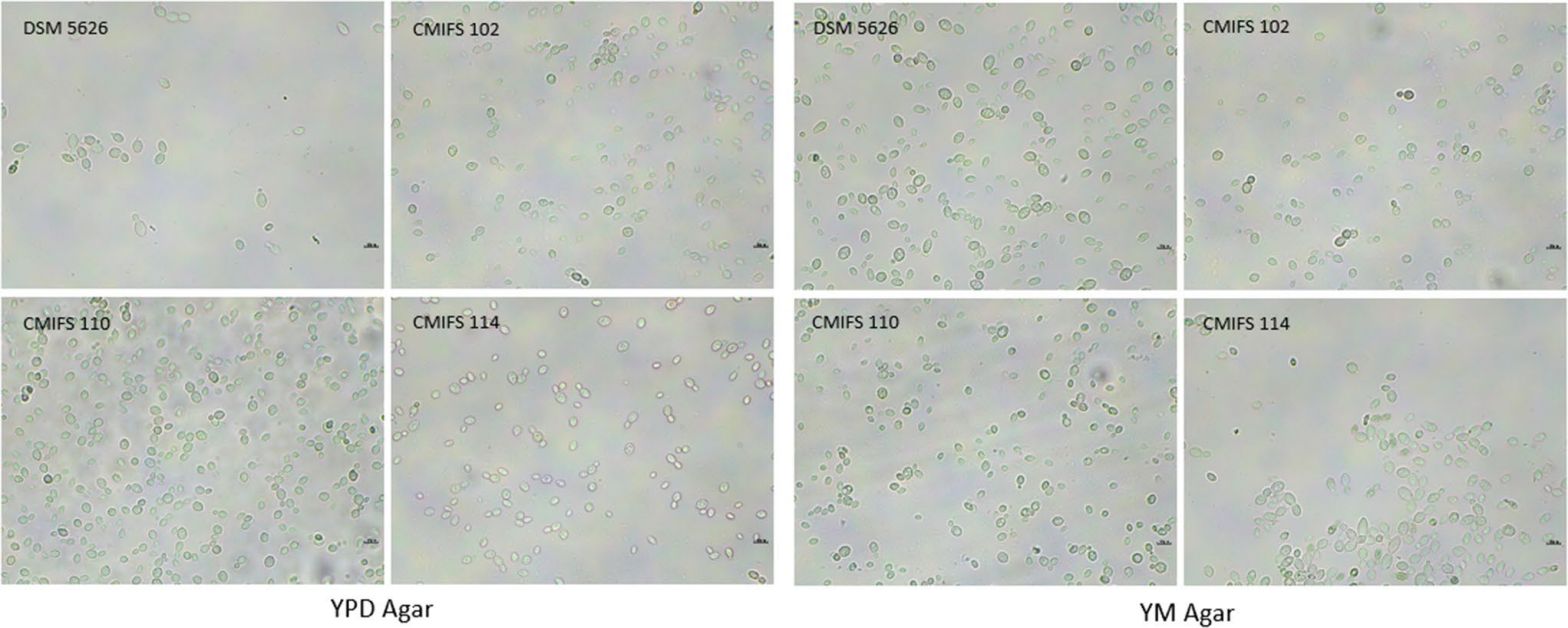
Morphology of the yeast *Phaffia rhodozyma* after cultivation on YPD and YM agar (20 °C/4 days). The photos were taken using a microscope Nexcope NE610 and a DLT-Camera Delta Optical (scale bars correspond to 10 μm)

After incubation the reference strain and isolates on YPD and YM nutrient media, the cells displayed an ovoid to spherical shape and reproduced asexually by polar budding (Fig. 3). The cells appeared singly, in pairs, or, less frequently, in very short chains. The reference strain DSM 5626 was characterized by slightly larger cell sizes (3.7–6.4 × 5.9–12.1 μm on YPD and 4.1–7.0 × 6.5–11.4 μm on YM) compared to the isolates: CMIFS 102 (3.2–6.1 × 4.8–10.4 μm on YPD and 3.5–6.5 × 6.1–10.8 μm on YM), CMIFS 110 (3.2–6.0 × 5.1–11.6 μm on YPD and 3.9–6.4 × 6.3–10.2 μm on YM), and CMIFS 114 (3.3–5.7 × 5.2–11.3 μm on YPD and 3.8–6.2 × 6.3–11.1 μm on YM).

**Fig. 4:**
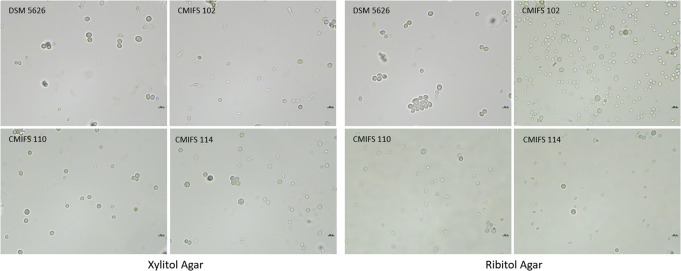
Morphology of the yeast *Phaffia rhodozyma* after cultivation on xylitol and ribitol agar (20 °C/14 days). The photos were taken using a microscope Nexcope NE610 and a DLT-Camera Delta Optical (scale bars correspond to 10 μm)

The capacity for sexual reproduction was assessed in polyol-containing media. According to Golubev (1995), polyols, especially ribitol, are the main factors in initiating sporulation in the yeast *Xanthophyllomyces dendrorhous* (the teleomorph of *P. rhodozyma*). Sexual structures form following the conjugation of the mother cell and its bud in the process of pedogamy, resulting in the formation of a slender holobasidium that contains three or four spores at the apex of the basidium (Libkind et al. 2008; David-Palma et al. 2016). As expected, the reference strain DSM 5626 (CBS 5905) did not produce sexual structures, consistent with observations by other researchers (Kucsera et al. 1998; Fell and Blatt 1999; Libkind et al. 2008). Similarly, no sexual reproduction was observed in any of the three isolates from Poland after cultivation on ribitol and xylitol media (Fig. 4). In addition to a few oval or elongated cells where budding was observed, numerous round structures larger than vegetative cells were noted, which strongly refracted light. These structures are probably chlamydospores, thick-walled, asexual spores that enable the microorganism to survive unfavorable environmental conditions. These forms are produced as a result of the compaction of protoplasm (Golubev 1995).

**Fig. 5:**
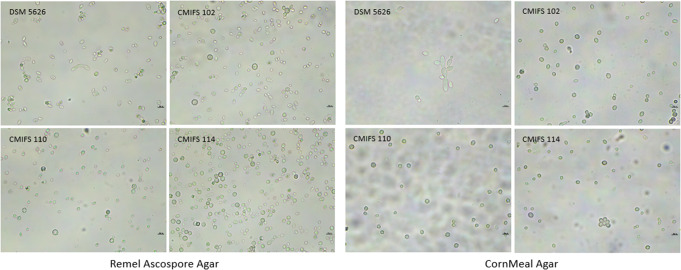
Morphology of the yeast *Phaffia rhodozyma* after cultivation on Remel Ascospore agar (20 °C/30 days) and CornMeal agar (20 °C/14 days). The photos were taken using a microscope Nexcope NE610 and a DLT-Camera Delta Optical (scale bars correspond to 10 μm)

The presence of chlamydospores was also detected after cultivating all strains on Remel Ascospore Agar. In addition, oval and spherical cells, as well as budding, were observed (Fig. 5). CornMeal Agar was used to study the formation of mycelium or pseudomycelium. Mycelium was not detected in any of the tested yeast strains. Only the reference strain produced pseudomycelium. For the remaining strains, spherical chlamydospores and ovoid budding cells were observed.

### Physiology

A special feature of *P. rhodozyma* that distinguishes this microorganism from other red yeasts is its ability to ferment sugars to ethanol under anaerobic conditions (Martínez-Cárdenas et al. 2018). All strains tested in this study were capable of fermenting glucose, fructose, and sucrose, but did not ferment galactose, lactose, maltose, or inulin. Isolates CMIFS 102 and 114 also weakly fermented trehalose and raffinose, while strains DSM 5626 and CMIFS 110 did not exhibit this property (Table 2). David-Palma et al. (2020) tested the fermentation abilities of *P. rhodozyma* CBS 7918 and CBS 5905 strains with various carbon sources. These strains also did not ferment galactose, maltose, lactose, or inulin, but did ferment glucose, mannitol, citric acid, succinic acid, and D-gluconic acid.

**Table 2:** Fermentation properties and the ability to assimilate various carbon and nitrogen compounds by *Phaffia Rhodozyma* yeast isolates compared to the reference strain

	Phaffia Rhodozyma**	*Phaffia Rhodozyma* DSM 5626	*Phaffia Rhodozyma* CMIFS 102	*Phaffia Rhodozyma* CMIFS 110	*Phaffia Rhodozyma* CMIFS 114
**Fermentation**					
D-Glucose	+	+	+	+	+
D-Fructose	n	+	+	+	+
D-Galactose	−	−	−	−	−
D-Sucrose	+	+	+	+	+
D-Lactose	−	−	−	−	−
D-Maltose	−/ws	−	−	−	−
D-Trehalose	w	−	w	−	w
D-Raffinose	w/−	−	w	−	w
Inulin	n	−	−	−	−
**Assimilation**					
D-Glucose	+	+	+	+	+
Glycerol	s/w	w	w	+	+
Calcium 2-ketogluconat	+	+	+	+	+
L-Arabinose	+	+	+	+	+
D-Xylose	+/s	+	−	+	+
Rybitol	n	+	+	+	+
Xylitol	w/−	−	−	+	+
D-Galactose	−	−	−	−	−
Inositol	−	−	−	−	−
D-Sorbitol	n	+	+	+	+
Methyl-α,D-glucopyranoside	w/−	−	−	−	−
N-acetylglucosamine	−	−	−	−	−
D-Cellobiose	+	+	+	+	+
DL-Lactate	−	−	−	−	−
D-Maltose	+	+	+	+	+
D-Sucrose	+	+	+	+	+
D-Trehalose	+	+	+	+	+
Melezitose	+	+	+	+	+
D-Raffinose	+/s	+	+	+	+
D-Fructose	n	+	+	+	+
DL-Lactate	v	−	−	−	−
Citric acid	+	+	+	−	−
D-Mannitol	+	+	+	+	+
Erythritol	−	−	−	−	−
Inulin	w/−	+	+	+	+
Nitrate	−	−	−	−	−
Ammonium sulfate	n	+	+	+	+
Urea	n	+	−	+	+

**Typical strains from The Yeasts: A Taxonomic Study— ed. Kurtzman C.P., Fell J.W. and Boekhout T. (2011); +—positive; l—latent (rapid development of a positive reaction after a lag period); s—positive but slow; w—weak; ws—weak and slow; lw—latent but weak (rapid development of a weak reaction after a lag period); v—variable; – negative; n—no data

All tested yeast strains were able to assimilate monosaccharides (glucose, arabinose, and fructose), disaccharides (maltose and sucrose), polysaccharides (cellobiose, trehalose, melezitose, raffinose, and inulin), sugar alcohols (glycerol, ribitol, sorbitol, and mannitol), and calcium 2-keto-D-gluconate. None of the strains assimilated galactose, inositol, methyl-α-D-glucopyranoside, N-acetylglucosamine, lactose, lactic acid, or erythritol. Differences were observed among strains for xylose, xylitol, and citric acid. The yeast *P. rhodozyma* CMIFS 102 did not metabolize xylose. Only strains DSM 5626 and CMIFS 102 utilized citric acid as a carbon source (Table 2). According to “The Yeasts: A Taxonomic Study,” typical strains of *P. rhodozyma* can metabolize citric acid (Fell and Johnson 2011). Additionally, the *P. rhodozyma* CBS 7918 and CBS 5905 strains tested by David-Palma et al. (2020) also can assimilate citrate. Conversely, for xylitol, the situation was different. Isolates CMIFS 110 and 114 were able to metabolize xylitol, while typical strains of *P. rhodozyma* either do not use this compound at all or metabolize it weakly (Fell and Johnson 2011).

All strains assimilated ammonium sulfate. However, only *P. rhodozyma* CMIFS 102 did not metabolize urea as a nitrogen source. None of the tested strains used nitrates (Table 2). Natesan and Gopal (2011) found that the yeast *P. rhodozyma* WG 07, isolated from tree leaves in India, demonstrated the ability to assimilate not only ammonium sulfate (VI) but also urea and ammonium nitrate. This proves that the origin of the strain determines its metabolic capabilities.

All strains produced starch-like compounds. There was no growth of *P. rhodozyma* strains in the vitamin-free medium (Table 3). This is consistent with the observations of Miller et al. (1976), who noted that *P. rhodozyma* cells require the addition of a vitamin source to the medium, including biotin, for growth.

**Table 3:** Selected physiological properties of yeast isolates and the reference strain of *Phaffia rhodozyma*

	*Phaffia rhodozyma* DSM 5626	*Phaffia rhodozyma* CMIFS 102	*Phaffia rhodozyma* CMIFS 110	*Phaffia rhodozyma* CMIFS 114
Starch formation	+	+	+	+
Vitamin-free	−	−	−	−
**Growth temperatures**				
4 °C	s	s	s	s
15 °C	+	+	+	+
20 °C	+	+	+	+
25 °C	+	+	+	+
30 °C	−	−	−	−
35 °C	−	−	−	−
**Growth at different pH**				
pH 1.0	−	−	−	−
pH 2.0	−	−	−	−
pH 3.0	s	s	s	s
pH 4.0	+	+	+	+
pH 5.0	+	+	+	+
pH 6.0	+	+	+	+
pH 7.0	+	+	+	+
pH 8.0	+	+	+	+
pH 9.0	+	+	+	+
pH 10.0	+	+	+	+
pH 11.0	+	+	+	+
pH 12.0	+	+	+	+
**Glucose resistance**				
5% glucose	+	+	+	+
10% glucose	+	+	+	+
20% glucose	+	+	+	+
30% glucose	+	+	+	+
40% glucose	+	+	+	+
50% glucose	s	s	s	s
**Sodium chloride resistance**				
0.5%	+	+	+	+
1%	+	+	+	+
2%	+	+	+	+
5%	+	+	+	+
7.5%	+	−	+	+
10%	−	−	−	−
15%	−	−	−	−
**Enzymatic activity**				
Urease	−	−	−	−
Gelatin liquefaction	−	−	−	−
Amylolytic enzymes	−	−	−	−
Lipolytic activity	−	−	−	−
Catalase	+	+	+	+
Cellulolytic enzymes	−	−	−	−

s—positive but slow

The growth of *P. rhodozyma* DSM 5626, CMIFS 102, 110, and 114 yeast cells was observed at 4, 15, 20, and 25 °C. At the highest temperatures (30 and 35 °C), these yeasts did not proliferate. David-Palma et al. (2020) showed that *Phaffia* yeast isolated from Australia grew at 25 °C but did not grow at 30 °C. Miller et al. (1976) reported that these psychrophilic yeasts can grow within a temperature range of 0–27 °C. Miao et al. (2021) confirmed that the inhibition of *Phaffia* yeast growth at temperatures above 28 °C is due to the suppression of DNA, RNA, fatty acid, and cell wall component biosynthesis.

A common feature of all strains is their ability to grow across a wide pH range (3.0–12.0). It has been shown that all tested yeast strains can grow in a medium containing 5–50% glucose. Tognetti et al. (2013) conducted a similar study on five strains of *P. rhodozyma* isolated in Argentina (CBS 7918T, CBS 5908, CBS 6938, CBS 7919, PYCC 4172, and CBS 5905T). The authors found that all strains grew in media with glucose concentrations of 20%, 30%, and 40%. Growth was inhibited or very weak in media with 50% glucose.

The tolerance of the tested yeasts to sodium chloride concentration in the medium reached 7.5%. The exception was the *P. rhodozyma* CMIFS 102 strain, which showed growth in a medium with a maximum NaCl concentration of 5.0%. Miller et al. (1976) reported that typical *P. rhodozyma* strains do not grow in media with 10% sodium chloride, a finding also confirmed in this work.

The results of the analysis of the ability to produce catalase, urease, amylolytic enzymes, cellulolytic enzymes, and gelatin liquefaction were identical for all four strains. Also the results of lipolytic activity (tests with tributyrin and Tween 80) were identical. The tested yeasts produced only catalase, with no activity detected for the other enzymes. The ability of *P. rhodozyma* to produce catalase is one of the mechanisms for protecting cells against reactive oxygen species Horváth et al. (2011). None of the strains produced amylolytic enzymes on starch. However, *P. rhodozyma* and representatives of the teleomorphic form *X. dendrorhous* may have this ability (Bhatt et al. 2013; Amado and Vázquez 2015). Díaz et al. (2003) found that the *P. rhodozyma* CECT 1690 strain synthesizes beta-amylase with a molecular weight of 240 kDa in the presence of starch and maltose in the medium.

### Influence of temperature on the biosynthesis of carotenoids

The cultivation temperature significantly influenced the growth of all yeast strains. The lowest cell biomass yields for all strains were obtained after 96 h of cultivation at temperatures of 12 16 and 24 °C (Table 4). For example, the *P. rhodozyma* CMIFS 114 strain produced 54% less cell biomass (2.88 g_d.m._/L) at 24 °C compared to 20 °C (6.34 g_d.m._/L). The maximum growth of the *P. rhodozyma* DSM 5626 strain was observed at temperatures between 18 and 22 °C (11.86–12.49 g_d.m._/L). Among the strains, the highest biomass was obtained from *P. rhodozyma* CMIFS 102 (9.27 g_d.m._/L) at 20 °C. The least biomass was produced by strains CMIFS 110 and 114, with amounts of 4.02 and 3.47 g_d.m._/L at 12 °C, and 5.59 and 4.21 g_d.m._/L at 16 °C, respectively. Guo et al. (2010) also investigated the effect of different temperatures (18, 20, 22, and 24 °C) on the biomass yield of the *P. rhodozyma* CGMCC As 2.1557 strain. Consistent with this work, the lowest biomass yield was recorded at 24 °C (5.1 g_d.m._/L), while yields at 18–20 °C were 7.4–7.6 g_d.m._/L. According to work (Miao et al. 2021) the optimal growth temperature for *P. rhodozyma* is in the range of 17–21 °C. This optimal range is likely due to the fact that these yeasts are isolated from native environments characterized by low temperatures, making them psychrophiles.

**Table 4:** Biomass yield, carotenoid content, and volumetric yield of carotenoids after 96 h of *Phaffia rhodozyma* cultivation at various temperatures

Parameter	Strain	Temperature					
		12 °C	16 °C	18 °C	20 °C	22 °C	24 °C
Biomass yield (g_d.m._/L)	DSM 5626	7.97 ± 0.75^c A^	10.37 ± 0.98^ab A^	12.49 ± 1.19^a A^	12.05 ± 0.71^a A^	11.86 ± 0.50^a A^	9.42 ± 1.18^b A^
	CMIFS 102	6.17 ± 0.49^c B^	7.19 ± 0.42^bc B^	8.48 ± 0.17^ab B^	9.27 ± 0.68^a B^	8.04 ± 0.45^b B^	5.92 ± 0.68^c B^
	CMIFS 110	4.02 ± 0.36^c C^	5.59 ± 0.44^b C^	7.32 ± 0.35^a C^	7.47 ± 0.58^a C^	6.81 ± 1.04^a B^	4.30 ± 0.35^c C^
	CMIFS 114	3.47 ± 0.30^c C^	4.21 ± 0.21^b D^	6.25 ± 0.35^a D^	6.34 ± 0.65^a C^	4.87 ± 0.29^b C^	2.88 ± 0.31^c D^
Carotenoid content in biomass (µg/g_d.m._)	DSM 5626	296.01 ± 9.69^a B^	251.29 ± 7.46^b B^	211.61 ± 7.28^c D^	214.26 ± 11.37^c B^	196.10 ± 7.07^cd B^	175.84 ± 9.66^d B^
	CMIFS 102	356.15 ± 7.37^a A^	335.52 ± 17.48^ab A^	316.49 ± 11.56^b A^	302.81 ± 18.65^bc A^	270.83 ± 10.42^c A^	230.22 ± 11.39^d A^
	CMIFS 110	358.35 ± 20.44^a A^	341.33 ± 14.76^a A^	272.96 ± 10.12^b B^	275.06 ± 17.85^b A^	265.39 ± 19.88^b A^	227.05 ± 12.73^c A^
	CMIFS 114	318.86 ± 15.06^a AB^	271.42 ± 11.59^b B^	247.60 ± 7.11^bc C^	232.45 ± 8.42^c B^	174.84 ± 6.89^d B^	128.95 ± 7.70^e C^
Volumetric yield of carotenoids (mg/L)	DSM 5626	2.36 ± 0.30^a A^	2.61 ± 0.30^a A^	2.64 ± 0.28^a A^	2.58 ± 0.11^a A^	2.33 ± 0.17^a A^	1.66 ± 0.30^b A^
	CMIFS 102	2.19 ± 0.14^b A^	2.42 ± 0.24^ab AB^	2.68 ± 0.08^a A^	2.82 ± 0.38^a A^	2.18 ± 0.05^b AB^	1.36 ± 0.19^c A^
	CMIFS 110	1.44 ± 0.20^b B^	1.91 ± 0.22^a B^	2.00 ± 0.09^a B^	2.05 ± 0.18^a B^	1.81 ± 0.35^ab B^	0.98 ± 0.12^c B^
	CMIFS 114	1.11 ± 0.06^b B^	1.14 ± 0.04^b C^	1.55 ± 0.05^a C^	1.48 ± 0.20^a C^	0.85 ± 0.03^c C^	0.37 ± 0.02^c C^

Different superscript letters (a, b, c) within the same row or different superscript letters (A, B, C, D, E) within the same column indicate significant (*p* < 0.05) differences in means. The mean values were compared using ANOVA one-way analyses of variance and Tukey’s test. Abbreviations: g_d.m._/L – grams of dry matter per liter of medium; µg/g_d.m._ – micrograms of carotenoids per liter of medium

All tested yeast strains synthesized the most carotenoids at the lowest temperature (12 °C). The highest amounts of carotenoids were found in the biomass of *P. rhodozyma* strains CMIFS 102 and 110, with 356.15 and 358.35 µg/g_d.m._, respectively. In comparison, strain DSM 5626 synthesized 296.01 µg/g_d.m._ of these compounds under the same conditions. As the temperature increased, the yeast synthesized progressively fewer pigments. After cultivation at 24 °C, the carotenoid content in the biomass of DSM 5626, CMIFS 102, CMIFS 110, and CMIFS 114 was reduced by 40%, 35%, 36%, and 59%, respectively, compared to cultivation at 12 °C. However, when considering cell biomass yields, the highest values for volumetric efficiency of carotenoid biosynthesis were obtained for the CMIFS strains at temperatures of 18 and 20 °C, and the DSM 5626 strain at temperatures between 16 and 20 °C. The highest value of this indicator was found after cultivating the *P. rhodozyma* CMIFS 102 isolate at 20 °C (2.82 mg/L) (Table 4). Guo et al. (2010) also determined the effect of different temperatures on the efficiency of astaxanthin biosynthesis by the yeast *P. rhodozyma* CGMCC As 2.1557. For the tested temperatures, the following results were obtained: 2.41 mg/L at 18 °C, 3.25 mg/L at 20 °C, 2.78 mg/L at 22 °C, and 1.98 mg/L at 24 °C. The volumetric efficiency of carotenoid biosynthesis at 20 °C was similar to that of the *P. rhodozyma* CMIFS 102 strain. A reduction in the efficiency of carotenoid biosynthesis was also observed at 24 °C, confirming that for these yeasts, the key parameter determining the carotenogenesis process is the selection of an appropriately low temperature.

### Influence of carbon source on carotenoid biosynthesis

The growth of the tested *P. rhodozyma* yeast strains depended on the carbon source used. The DSM 5626 reference strain effectively utilized all tested compounds (9.31–12.32 g_d.m._/L), except for glycerol (4.08 g_d.m._/L). The *P. rhodozyma* CMIFS 102 isolate showed the highest biomass yield in the medium with maltose (13.81 g_d.m._/L), followed by sucrose (9.43 g_d.m._/L) and glucose (9.27 g_d.m._/L). Similar to the DSM 5626 strain, a significant decrease in biomass yield was observed after culturing in a medium with glycerol (2.21 g_d.m._/L). The CMIFS 102 strain was the only one that did not assimilate xylose and, therefore, did not grow in this medium. Most strains of the *P. rhodozyma* species can metabolize xylose, which can be used to dispose of some waste. For example, Parajo et al. (2000) used a liquid medium containing xylose (16.6 g_d.m._/L) obtained from hemicellulose hydrolysates of *Eucalyptus globulus* wood and 3 g of peptone/L as a carbon source. After cultivation in this medium, 10.5 g of dry substance of the yeast *P. rhodozyma* NRRL Y-17,268 was obtained from one liter of medium. Natesan and Gopal (2011) conducted research allowing for the comparison of biomass yield results for glucose, maltose, sucrose, and mannitol. They cultivated *P. rhodozyma* WG 07 and obtained biomass yield values of 11.0 g_d.m._/L (glucose), 12.5 g_d.m._/L (maltose), 14.3 g_d.m._/L (sucrose), and 9.8 g_d.m._/L (mannitol). The biomass yield values in the maltose medium were almost identical to those obtained for the CMIFS 102 strain. However, in the other media, the *P. rhodozyma* WG 07 strain, originally isolated from tree leaves in India, showed better growth.

For the CMIFS 110 isolate, the highest yield (11.10 g_d.m._/L) was obtained after cultivation in a sucrose medium. This strain also efficiently utilized maltose (9.25 g_d.m._/L) and fructose (8.22 g_d.m._/L). Interestingly, the CMIFS 110 isolate was the only one to effectively use glycerol (7.11 g_d.m._/L), with the biomass yield being similar to that obtained after cultivation in a medium with glucose (7.47 g_d.m._/L). The lowest yield was observed after cultivation in a medium with molasses (4.46 g_d.m._/L). The ability to metabolize glycerol as a carbon source can be utilized to process the glycerol fraction after biodiesel production.Kusdiyantini et al. (1998) cultivated the *P. rhodozyma* PR 190 yeast in a medium initially containing 2.8% glycerol as a carbon source, with constant pH adjustment to 6.0, and obtained 18.27 g of biomass from 1 L of medium.

Similar to the DSM 5626 and CMIFS 110 strains, the *P. rhodozyma* CMIFS 114 yeast showed the highest biomass yield after cultivation in a sucrose medium (9.07 g_d.m._/L). This strain also effectively used molasses (8.39 g_d.m._/L) and fructose (7.51 g_d.m._/L), while it showed the lowest yields after cultivation in media with glycerol (3.81 g_d.m._/L) and xylose (4.12 g_d.m._/L). The effective use of molasses for the proliferation of *P. rhodozyma* cells was also reported by Kanwugu et al. (2020). They cultivated the yeast *P. rhodozyma* Y1654 in media with soy or sugar beet molasses. Media based on soy molasses resulted in the highest biomass yield (7.7 g_d.m._/L), while cultivation in a medium with sugar beet molasses yielded a lower value of 5.8 g_d.m._/L.

The yeast *P. rhodozyma* DSM 5626 synthesized the most carotenoids when grown in media with mannitol (262.25 µg/g_d.m._), fructose (257.34 µg/g_d.m._), and sucrose (249.85 µg/g_d.m._) (Table 5). The addition of glycerol as a carbon source not only limited the growth of this strain but also its ability to biosynthesize carotenoids (66.53 µg/g_d.m._). The volumetric efficiency of carotenoid biosynthesis was highest after cultivation in media with sucrose and fructose (3.07–3.08 mg/L). Similar values for this indicator in the yeast *P. rhodozyma* CGMCC As 2.1557 in media containing sucrose and fructose were reported by Guo et al. (2010). The addition of fructose to the medium resulted in 2.92 mg of astaxanthin/L, while sucrose led to 3.22 mg/L.

**Table 5:** Biomass yield, carotenoid content, and volumetric yield of carotenoids after 96 h of *Phaffia rhodozyma* cultivation in media with various carbon sources

Parameter	Strain	Carbon source							
		Glucose	Fructose	Xylose	Maltose	Sucrose	Glycerol	Mannitol	Molasses
Biomass yield (g_d.m._/L)	DSM 5626	12.05 ± 0.71^a A^	11.91 ± 0.57^a A^	10.48 ± 0.20^b A^	12.22 ± 0.69^a A^	12.32 ± 0.31^a A^	4.08 ± 0.49^d B^	9.31 ± 0.62^c A^	9.49 ± 0.40^c A^
	CMIFS 102	9.27 ± 0.68^b B^	8.30 ± 0.28^b B^	0.99 ± 0.31^e D^	13.81 ± 0.89^a A^	9.43 ± 0.25^b C^	2.21 ± 0.29^d C^	4.91 ± 0.53^c B^	8.33 ± 0.16^b A^
	CMIFS 110	7.47 ± 0.58^c C^	8.22 ± 0.52^bc B^	5.69 ± 0.27^d B^	9.25 ± 0.36^b B^	11.10 ± 0.24^a B^	7.11 ± 0.29^c A^	5.78 ± 0.08^d B^	4.46 ± 0.56^e B^
	CMIFS 114	6.34 ± 0.65^bc C^	7.51 ± 0.45^b B^	4.12 ± 0.43^d C^	5.68 ± 0.27^c C^	9.07 ± 0.34^a C^	3.81 ± 0.14^d B^	5.66 ± 0.41^c B^	8.39 ± 0.23^ab A^
Carotenoid content in biomass (µg/g_d.m._)	DSM 5626	214.26 ± 11.37^c B^	257.34 ± 14.43^a B^	206.34 ± 5.30^c A^	218.99 ± 12.20^c C^	249.85 ± 9.12^ab B^	66.53 ± 5.63^d D^	262.25 ± 16.38^a A^	238.31 ± 9.83^b A^
	CMIFS 102	302.45 ± 18.65^a A^	295.43 ± 4.80^a A^	0.00 ± 0.00^e C^	260.61 ± 10.45^b B^	290.61 ± 10.86^a A^	129.44 ± 9.93^d B^	213.16 ± 7.46^c B^	150.59 ± 20.23^d C^
	CMIFS 110	281.73 ± 15.18^ab A^	229.76 ± 17.75^c C^	169.17 ± 13.49^e B^	305.78 ± 13.32^a A^	272.99 ± 5.66^b A^	172.87 ± 9.82^de A^	235.20 ± 9.02^c AB^	190.28 ± 6.03^d B^
	CMIFS 114	232.45 ± 8.42^b B^	250.78 ± 8.57^a BC^	161.78 ± 11.71^d B^	227.77 ± 8.31^b C^	229.39 ± 8.03^b B^	91.36 ± 6.18^e C^	196.73 ± 9.67^c C^	180.22 ± 4.38^cd B^
Volumetric yield of carotenoids (mg/L)	DSM 5626	2.58 ± 0.11^bc A^	3.07 ± 0.27^a A^	2.16 ± 0.10^d A^	2.68 ± 0.25^b B^	3.08 ± 0.18^a A^	0.27 ± 0.02^e B^	2.44 ± 0.05^c A^	2.26 ± 0.14^d A^
	CMIFS 102	2.82 ± 0.38^b A^	2.45 ± 0.08^b B^	0.00 ± 0.00^e D^	3.59 ± 0.12^a A^	2.74 ± 0.16^b A^	0.29 ± 0.04^d B^	1.05 ± 0.15^c C^	1.25 ± 0.15^c C^
	CMIFS 110	2.11 ± 0.23^b B^	1.89 ± 0.25^b C^	0.96 ± 0.06^d B^	2.83 ± 0.10^a B^	3.03 ± 0.10^a A^	1.23 ± 0.04^c A^	1.36 ± 0.04^c B^	0.85 ± 0.13^d D^
	CMIFS 114	1.48 ± 0.20^c C^	1.88 ± 0.06^b C^	0.67 ± 0.12^f C^	1.29 ± 0.05^d C^	2.08 ± 0.01^a B^	0.35 ± 0.02^g B^	1.11 ± 0.04^e C^	1.51 ± 0.05^c B^

Different superscript letters (a, b, c, d, e, f, g) within the same row or different superscript letters (A, B, C, D) within the same column indicate significant (*p* < 0.05) differences in means. The mean values were compared using ANOVA one-way analyses of variance and Tukey’s test. Abbreviations: g_d.m._/L – grams of dry matter per liter of medium; µg/g_d.m._ – micrograms of carotenoids per liter of medium

Isolates obtained from birch slime fluxes synthesized carotenoids with different efficiency, depending on the strain and the carbon source used. For the yeast *P. rhodozyma* CMIFS 102, the highest carotenoid content was found in the biomass after cultivation in media with glucose (302.81 µg/g_d.m._), fructose (295.43 µg/g_d.m._), and sucrose (290.09 µg/g_d.m._). However, after converting the carotenoid content to a per-unit culture medium basis, the highest volumetric biosynthetic efficiency was observed in the medium with the addition of maltose (3.59 mg/L). Similarly to the DSM 5626 collection strain, isolate CMIFS 102 produced the least amount of carotenoids after cultivation in a glycerol medium (129.44 µg/g_d.m._). In the case of the *P. rhodozyma* CMIFS 110 strain, significantly higher carotenoid content in biomass and volumetric biosynthetic efficiency were recorded only after culturing with maltose (305.78 µg/g_d.m._; 2.83 mg/L) and sucrose (272.99 µg/g_d.m._; 3.03 mg/L). The ability of *P. rhodozyma* yeast to biosynthesize carotenoids more efficiently in media containing glucose, sucrose, and maltose as carbon sources enables the management of various food wastes. For example, research by Lai et al. (2022), used a medium prepared from peppers, skins, and meat bones obtained from a student canteen. After pretreatment, the concentrations of individual sugars in the medium were 2.2% glucose, 3.3% maltose, and 2.3% xylose. After optimizing the culture conditions, the *P. rhodozyma* Y119 yeast synthesized carotenoids with an efficiency of up to 129.5 mg/L.

Compared to the reference strain DSM 5626 and isolates CMIFS 102 and 110, the yeast *P. rhodozyma* CMIFS 114 synthesized carotenoids with the lowest efficiency (Table 5). The highest total carotenoid content was found in the biomass obtained after cultivation in a medium with fructose (250.78 µg/g_d.m._), while the highest volumetric yield was recorded after cultivation in a medium with sucrose (2.08 mg/L). Yamane et al. (1997) also found that fructose and sucrose are effective substrates for carotenoid biosynthesis by the *P. rhodozyma* ATCC 24,202 strain. After cultivation in liquid media with fructose and sucrose as the only carbon sources, the efficiency of astaxanthin biosynthesis was 2.14 and 2.09 mg/L, respectively.

### Influence of nitrogen source on carotenoid biosynthesis

After cultivating *P. rhodozyma* DSM 5626 yeast in media containing various nitrogen sources, the highest biomass yield (15.00 g_d.m._/L) was found in media containing deproteinized potato wastewater (Table 6). High biomass yields were also recorded in media with ammonium sulfate (12.05 g_d.m._/L) and casein hydrolyzate (11.34 g_d.m._/L). The nitrogen source least assimilable by this strain was urea (4.42 g_d.m._/L). Potato wastewater can be an excellent source of nitrogen for the growth of *Candida* (Kurcz et al. 2018), *Trichosporon* (Gientka et al. 2019), and *Rhodotorula* (Kot et al. 2019) yeasts. It is a byproduct generated during the production of potato starch. Additionally, Zhang et al. (2023) showed that deproteinized potato juice can serve as a substrate for the yeast *P. rhodozyma* SFAS-TZ08. After 120 h of cultivation in a bioreactor, 45.2 g of biomass were obtained from one liter of medium.

**Table 6:** Biomass yield, carotenoid content, and volumetric yield of carotenoids after 96 h of *Phaffia rhodozyma* cultivation in media with various nitrogen sources

Parameter	Strain	Nitrogen source						
		(NH_4_)_2_SO_4_	Peptone	Urea	(NH_4_)_2_HPO_4_	Casein hydrolyzate	Potato wastewater	NH_4_Cl
Biomass yield (g_d.m._/L)	DSM 5626	12.05 ± 0.71^b A^	10.77 ± 0.27^c B^	4.42 ± 0.44^e B^	10.40 ± 0.32^c A^	11.34 ± 0.31^bc B^	15.00 ± 0.28^a B^	7.98 ± 0.75^d A^
	CMIFS 102	9.27 ± 0.68^cd B^	12.38 ± 0.22^b A^	0.46 ± 0.15^e C^	10.62 ± 0.43^c A^	12.39 ± 0.25^b A^	18.36 ± 0.54^a A^	8.92 ± 0.38^d A^
	CMIFS 110	7.47 ± 0.58^cd C^	11.96 ± 0.46^b A^	5.79 ± 0.38^d A^	8.93 ± 0.41^c B^	11.48 ± 0.19^b B^	14.82 ± 0.58^a B^	6.18 ± 0.21^d B^
	CMIFS 114	6.34 ± 0.65^e C^	11.09 ± 0.27^b AB^	6.30 ± 0.16^e A^	8.09 ± 0.20^d B^	9.47 ± 0.35^c C^	13.62 ± 0.20^a B^	4.23 ± 0.09^f C^
Carotenoid content in biomass (µg/g_d.m._)	DSM 5626	214.26 ± 11.37^b B^	238.67 ± 13.88^ab A^	181.91 ± 7.21^c A^	207.01 ± 7.21^b B^	158.01 ± 13.54^d C^	224.32 ± 7.61^b B^	260.60 ± 8.75^a B^
	CMIFS 102	302.81 ± 18.65^a A^	268.60 ± 16.03^b A^	0.00 ± 0.00^d C^	252.63 ± 15.68^b A^	223.94 ± 7.83^c A^	262.20 ± 6.59^b A^	281.21 ± 4.53^ab AB^
	CMIFS 110	281.73 ± 15.18^ab A^	193.22 ± 9.40^c B^	113.40 ± 9.58^d B^	236.67 ± 8.35^b A^	188.76 ± 3.77^c B^	251.20 ± 8.61^b A^	296.20 ± 12.13^a A^
	CMIFS 114	232.45 ± 8.42^b B^	171.97 ± 8.37^c C^	170.23 ± 12.00^c A^	180.49 ± 7.38^c C^	152.62 ± 3.05^d C^	248.10 ± 14.57^b A^	299.27 ± 13.27^a A^
Volumetric yield of carotenoids (mg/L)	DSM 5626	2.58 ± 0.11^b AB^	2.57 ± 0.21^b B^	0.80 ± 0.05^e B^	2.15 ± 0.14^c B^	1.79 ± 0.15^d C^	3.37 ± 0.17^a B^	2.08 ± 0.16^c B^
	CMIFS 102	2.82 ± 0.38^bc A^	3.32 ± 0.16^b A^	0.00 ± 0.00^e D^	2.69 ± 0.27^cd A^	2.78 ± 0.15^c A^	4.81 ± 0.10^a A^	2.51 ± 0.11^d A^
	CMIFS 110	2.11 ± 0.23^b B^	2.31 ± 0.18^b BC^	0.65 ± 0.03^d C^	2.11 ± 0.15^b B^	2.17 ± 0.01^b B^	3.72 ± 0.20^a B^	1.83 ± 0.10^c B^
	CMIFS 114	1.48 ± 0.20^c C^	1.91 ± 0.07^b C^	1.07 ± 0.07^e A^	1.46 ± 0.09^c C^	1.45 ± 0.08^c D^	3.38 ± 0.25^a B^	1.26 ± 0.06^d C^

Different superscript letters (a, b, c, d, e, f) within the same row or different superscript letters (A, B, C, D) within the same column indicate significant (*p* < 0.05) differences in means. The mean values were compared using ANOVA one-way analyses of variance and Tukey’s test. Abbreviations: g_d.m._/L – grams of dry matter per liter of medium; µg/g_d.m._ – micrograms of carotenoids per liter of medium

The *P. rhodozyma* CMIFS 102 strain also produced the highest biomass after cultivation in a medium with potato wastewater (18.36 g_d.m._/L). Similar results were obtained after cultivation in media with peptone (12.38 g_d.m._/L) and casein hydrolyzate (12.39 g_d.m._/L). Significantly less biomass was produced by the yeast in media containing ammonium sulfate, ammonium dihydrogen phosphate, and calcium chloride (8.92–10.62 g_d.m._/L). The limited growth in the urea medium resulted from the CMIFS 102 strain’s inability to metabolize this compound (Table 2).

The yeast *P. rhodozyma* CMIFS 110 produced the most biomass when grown in media containing potato wastewater (14.82 g_d.m._/L), peptone (11.96 g_d.m._/L), and casein hydrolyzate (11.48 g_d.m._/L). The least effective substrates were ammonium chloride (6.18 g_d.m._/L) and urea (5.79 g_d.m._/L). The *P. rhodozyma* CMIFS 114 isolate produced the least biomass among the tested strains in all media except for urea, where the biomass yield was 6.30 g_d.m._/L. Similar to the other strains, the best nitrogen source for growth for the CMIFS 114 strain was potato juice water (13.62 g_d.m._/L), while ammonium chloride was the least effective source (4.23 g_d.m._/L). A similar biomass yield for the yeast *P. rhodozyma* 7B12 (4.95 g_d.m._/L) was obtained after cultivation in a medium with ammonium chloride as the only nitrogen source, as reported by Ni et al. (2007).

The *P. rhodozyma* DSM 5626 strain produced the highest carotenoid levels in media containing ammonium chloride (260.60 µg/g_d.m._) and peptone (238.67 µg/g_d.m._). Other suitable nitrogen sources included potato wastewater (224.32 µg/g_d.m._), ammonium sulfate (214.26 µg/g_d.m._), and diammonium hydrogen phosphate (207.01 µg/g_d.m._). Considering the amount of biomass obtained, the highest volumetric biosynthetic efficiency was achieved in the medium with potato wastewater, reaching 3.37 mg/L (Table 6). The lowest carotenoid yield was observed in the medium with casein hydrolyzate (1.79 mg/L). Interestingly, Ni et al. (2007) reported the highest level of carotenoid biosynthesis by the *P. rhodozyma* 7B12 strain in a casein hydrolyzate medium (5.62 mg/L). This indicates that selecting the appropriate substrate components is essential for maximizing carotenoid biosynthesis for each yeast strain.

The highest total carotenoid production among the tested yeasts was achieved by the *P. rhodozyma* CMIFS 102 strain. The highest carotenoid content in the biomass was observed in media with ammonium sulfate (302.81 µg/g_d.m._) and ammonium chloride (281.21 µg/g_d.m._) as nitrogen sources. However, after accounting for biomass yield, the highest volumetric biosynthetic efficiency was found in the medium with potato wastewater (4.81 mg/L), which is the highest efficiency achieved in this work. Similar to the work by Fang and Cheng (1993), where the *P. rhodozyma* NCHU-FS301 strain was tested, no carotenoid biosynthesis was observed in the medium containing urea as a nitrogen source.

Similarly to the CMIFS 102 strain, the *P. rhodozyma* CMIFS 110 yeast synthesized the highest amount of carotenoids in media with ammonium chloride (296.20 µg/g_d.m._) and ammonium sulfate (281.73 µg/g_d.m._). High content of these compounds was also observed in the biomass obtained from cultivation in a medium with potato wastewater (251.20 µg/g_d.m._), which, as with other strains, guaranteed the highest volumetric biosynthetic efficiency (3.72 mg/L). The same trend was found for the last tested isolate of *P. rhodozyma* CMIFS 114. The highest amount of carotenoids were found in biomass obtained from cultivation in media with ammonium chloride (299.27 µg/g_d.m._) and ammonium sulfate (232.45 µg/g_d.m._). However, due to the high biomass yield, the volumetric biosynthetic efficiency was greatest in the medium with potato wastewater (3.38 mg/L). These results prove that potato wastewater can be a cost-effective source of nitrogen, guaranteeing excellent growth and efficient carotenoid biosynthesis, as it also provides minerals and various vitamins. Zhang et al. (2023) found that after scaling up the culture from flasks to a 5-L bioreactor and cultivating *P. rhodozyma* SFAS-TZ08 yeast in a medium with potato juice and yeast extract for 120 h, they obtained 19.465 mg of carotenoids per liter of medium.

## Conclusions

The yeast *P. rhodozyma* has significant biotechnological potential due to its ability to synthesize astaxanthin, making it valuable in aquaculture. In this work, three new strains of *P. rhodozyma*, previously isolated from birch slime fluxes in Poland, were characterized. Phenotypic analysis revealed minor differences in their ability to assimilate xylose, xylitol, citric acid, and urea. These differences may be attributed to the geographical specificity of the habitat from which they were originally isolated. Analysis of the protein spectra of CMIFS strains using MALDI-TOF MS confirmed a high similarity to the spectra of the reference strain. However, the differences detected in the intensity and type of spectra indicate heterogeneity within the species. All strains synthesized carotenoids, with synthesis dependent on the type of carbon and nitrogen source in the medium as well as the culture temperature. Considering the volumetric efficiency of carotenoid biosynthesis, sucrose was the best carbon source, while deproteinized potato wastewater was the most effective nitrogen source. The information provided in this work increases knowledge about the biodiversity of *P. rhodozyma* and may be useful for future research assessing the potential of this yeast as a source of natural carotenoids.

## Data Availability

No datasets were generated or analysed during the current study.
